# Supplementary material for: Multi‐dataset identification of innovative feature genes and molecular mechanisms in keratoconus
Source: J Cell Mol Med. 2024 Sep 19;28(18):e70079. doi: 10.1111/jcmm.70079 (PMC11412914; doi:10.1111/jcmm.70079)
Supplement: Supplementary file 2 — Material S2. [file JCMM-28-e70079-s002.zip › 7.GSEA analysis/4.MYRF/KEGG_TERPENOID_BACKBONE_BIOSYNTHESIS.html]

Details for gene set KEGG\_TERPENOID\_BACKBONE\_BIOSYNTHESIS[GSEA]

|  || Dataset | MYRF.group.cls#h\_versus\_l |
| Phenotype | group.cls#h\_versus\_l |
| Upregulated in class | h |
| GeneSet | KEGG\_TERPENOID\_BACKBONE\_BIOSYNTHESIS |
| Enrichment Score (ES) | 0.44499883 |
| Normalized Enrichment Score (NES) | 1.1979862 |
| Nominal p-value | 0.2772074 |
| FDR q-value | 1.0 |
| FWER p-Value | 0.987 |
Table: GSEA Results Summary

  

Fig 1: Enrichment plot: KEGG\_TERPENOID\_BACKBONE\_BIOSYNTHESIS      
 Profile of the Running ES Score & Positions of GeneSet Members on the Rank Ordered List

  

| SYMBOL | TITLE | RANK IN GENE LIST | RANK METRIC SCORE | RUNNING ES | CORE ENRICHMENT || 1 | MVD | na | 5719 | 0.201 | 0.0104 | Yes |
| 2 | MVK | na | 7199 | 0.158 | 0.1060 | Yes |
| 3 | PDSS1 | na | 9252 | 0.110 | 0.1432 | Yes |
| 4 | HMGCR | na | 10147 | 0.093 | 0.1988 | Yes |
| 5 | FDPS | na | 10800 | 0.082 | 0.2512 | Yes |
| 6 | IDI1 | na | 11270 | 0.074 | 0.3021 | Yes |
| 7 | HMGCS2 | na | 11634 | 0.069 | 0.3515 | Yes |
| 8 | ACAT2 | na | 12097 | 0.062 | 0.3923 | Yes |
| 9 | PMVK | na | 12132 | 0.062 | 0.4450 | Yes |
| 10 | HMGCS1 | na | 15227 | 0.026 | 0.3782 | No |
| 11 | ACAT1 | na | 17648 | 0.003 | 0.3110 | No |
| 12 | GGPS1 | na | 18070 | -0.001 | 0.2998 | No |
| 13 | IDI2 | na | 22754 | -0.056 | 0.2135 | No |
| 14 | PDSS2 | na | 23173 | -0.062 | 0.2558 | No |
| 15 | DHDDS | na | 24723 | -0.088 | 0.2882 | No |
Table: GSEA details [plain text format]

  

Fig 2: KEGG\_TERPENOID\_BACKBONE\_BIOSYNTHESIS      
 Blue-Pink O' Gram in the Space of the Analyzed GeneSet

  

Fig 3: KEGG\_TERPENOID\_BACKBONE\_BIOSYNTHESIS: Random ES distribution      
 Gene set null distribution of ES for **KEGG\_TERPENOID\_BACKBONE\_BIOSYNTHESIS**
